# Smart TPE Materials Based on Recycled Rubber Shred

**DOI:** 10.3390/ma14216237

**Published:** 2021-10-20

**Authors:** Klaudia Toczek, Magdalena Lipińska, Joanna Pietrasik

**Affiliations:** Institute of Polymer and Dye Technology, Lodz University of Technology, Stefanowskiego 16, 90-537 Lodz, Poland; klaudia.toczek@dokt.p.lodz.pl (K.T.); joanna.pietrasik@p.lodz.pl (J.P.)

**Keywords:** shape memory materials, material recycling, ethylene-1-octene thermoplastic elastomers, rubber shreds, polymer blends

## Abstract

Thermo-responsive shape memory materials were developed based on recycled ethylene-propylene-diene (EPDM) rubber shred and thermoplastic elastomers (TPE). Ethylene-1-octene TPEs (Engage 8180, 8411, 8452) with varying degrees of crystallinity and Mooney viscosity were used to prepare the composite materials. To avoid the deterioration of static mechanical properties after mixing recycled EPDM rubber shred (RS) with thermoplastic elastomers, they were partially cured using dicumyl peroxide. The peroxide curing was the most effective for a rubber shred/Engage 8180 blend, where the highest cure rate index (CRI), 1.88 dNm⋅min^−1^, was observed. The curing caused an approximately 4-fold increase of tensile strength (TS) values for EPDM rubber shred/thermoplastic elastomer blend to the level acceptable for the rubber industry compared with an uncured blend. The incorporation of EPDM rubber shred changed thermoplastic elastomers’ viscoelastic behavior, increasing the values of storage (G′) and loss (G″) modulus. The lowest viscosity of molten Engage 8411 during mixing led to higher compatibility of rubber shred RS/8411 blend, as confirmed by analysis of Cole-Cole plots and the blend morphology. All rubber shred RS/TPE blends showed the shape memory behavior. For the RS/Engage 8452 blend, the highest shape fixity (F) value (94%) was observed, while the shape recovery (RR) was 87%. Studies confirmed that the intelligent materials with shape memory effect could be obtained via selectively chosen thermoplastic elastomers; ethylene-1-octene as a binder for recycled EPDM. Prepared recycled TPE/rubber shred blends can be successfully reused due to their viscoelastic and mechanical properties. Therefore, such a concept can be potentially interesting for the rubber industry.

## 1. Introduction

The production of rubber and plastics products has been growing rapidly, but at the same time, the amount of waste also increases [1]. Rubber is produced by crosslinking of elastomers and is widely used in industry due to its versatile properties. However, it is difficult to recycle rubber waste or discard it, using the methods generally employed for thermoplastic materials due to the presence of a three-dimensional structure resulting from the so-called crosslinked system. Composting of rubber waste is also a big problem, as rubber is not biodegradable and harms the environment [2,3].

Ethylene-propylene-diene (EPDM) rubber is one of the most often used synthetic rubbers due to its very good resistance to weather conditions, heat, oxygen, and ozone [4]. Common EPDM waste recycling methods are based on devulcanization techniques that assume that the crosslinks are partially destroyed by chemical, thermochemical or microwave-chemical processes [5,6,7]. The disadvantages of the mentioned processes result from the fact that the crosslinks are cleaved and partly the polymer chains. This leads to the formation of branched macromolecules with a molecular weight distribution broader than that of the original elastomer. Thus, it can cause a deterioration of mechanical properties for the devulcanized material. Additionally, the accelerators remaining in the waste rubber also play a negative role in the waste rubber recycling process [5]. Among other potential commercial methods, the mechanical defragmentation of rubber products into small pieces (rubber shred), similar to the mechanical recycling used for thermoplastic waste, seems to be the most promising [8]. Furthermore, [9,10,11,12] demonstrated that EPDM rubber shred could be added to other polymers or elastomers.

The immiscibility of mechanical recycled EPDM with other rubbers is the main reason leading to the deterioration of the mechanical properties of blends [9]. It is well known that the mechanical properties of blends can be significantly improved by the enhancement of the compatibility between phases. Nabil et al. [10] found that effective compatibility between the natural rubber/EPDM/recycled-EPDM blends could be generated after the addition of carbon black and the preheating of EPDM/recycled-EPDM to 150 °C before mixing it with natural rubber (NR). This reactive processing method led to more homogenous morphology of the blend, higher crosslink density, and better carbon black dispersion leading to the enhancement of mechanical properties. Pre-vulcanization and electron beam irradiation were also used to improve the rubber blends’ compatibility with recycled EPDM [11]. Mixing ground EPDM waste with natural rubber latex before compounding with dry rubber using a two-roll-mill (a two-steps method) was also found to improve the properties of recycled EPDM blends [12]. Herein, the ground EPDM insulation materials waste was added to ethylene-1-octene thermoplastic elastomers. As a result, smart shape memory materials were generated.

Shape memory polymeric blends belong to the class of smart materials (SM) due to their ability to detect and respond to external stimuli, e. g. temperature, light, electric field can change their properties such as shape, color, electrical conductivity, etc. [13]. Shape memory polymers (SMPs) represent a promising class of smart materials [13,14,15,16,17]. SMPs change their shape in a predefined manner from a less constrained shape to a temporary constrained shape and then return to their original shape again when restored by an external stimulus [13]. Depending on the external stimulus to which the SMP responds, SMPs may fall into different categories: thermally reactive (temperature-dependent), chemically reactive (chemicals, e. g. water, ethanol), pH-dependent, photosensitive (light dependent), electrically reactive (electricity-dependent), or magnetically reactive (magnetic field dependent) [13,15]. The first shape memory polymer material, known since the 1960s, was radiation crosslinked polyethylene (PE-X), which is still commercially used in the form of heat-shrinkable tubing [18]. SMPs have found applications in a variety of fields, such as cable and packaging industry [19], medical and automotive [19,20], in smart textiles and clothing [15,21], in heat-shrink packaging for electronics [22], sensors and actuators [15,23,24], high water vapor permeability materials [15,25], self-degradable structures in spacecraft [21].

Thermally reactive smart polymeric materials are one of the most popular [26]. The appearance of thermally induced effect requires the presence of crosslinks (covalent bonds), hydrogen or ionic bonds, as well as physical intermolecular interactions between polymers [19,26] and phase transition (melting) at convenient T_trans_ temperatures [19,20,26]. The crystalline phase formed during the cooling of the programmed sample can effectively fix the deformation and viscoelastic forces accumulated in the lattice [19]. Although the glass transition temperature (T_g_) or melting temperature (T_m_) of the soft segment can be the transition temperature (T_trans_) for these types of blends, the melting temperature is preferable because it can better determine the shape recovery temperature of the blends [26,27,28,29]. Upon high-temperature deformation (T > T_trans_), the materials transform into a temporary shape in which the chain segments disentangle and elongate, and this temporary and dormant shape can be fixed by cooling below the transition temperature (T < T_trans_) [26,27,28,29]. Again, triggering a higher temperature (T > T_trans_) allows the material to return to its original shape. Thermally-induced SM behavior is described using factors: recovery ratio (RR) and shape fixity (F) [30]. 

Currently, the structural design of SMPs can be achieved by grafting [31], copolymerization [14,32], formation of interpenetrating polymer networks (IPNs) [33,34], or blending of polymers and rubbers [35,36,37,38,39]. They all aim to obtain a multiphase material exhibiting at least two different transition temperatures. Among the above preparation techniques, mixing two immiscible polymers is very often used because it is an effective, convenient, and economical method to obtain a mixture with a two-phase structure and shape memory properties.

It was reported [38] that ethylene-propylene-diene rubber/polypropylene EPDM/PP blends prepared by two-roll mixing and further crosslinked by peroxide-induced dynamic vulcanization showed shape memory behavior. The polypropylene phase allowed one to fix a temporary shape, while the EPDM phase acted as the driving force for shape recovery. EPDM/PP blends with an interface modified by the addition of magnesium acrylate showed the shape fixity ~90% and shape recovery ~92.5% [38]. Thermoplastic polymers such as polyethylene [36] or polypropylene [37] were directly mixed with thermoplastic elastomers to obtain shape memory materials. Moreover, polyethylene/polycyclooctene blends crosslinked by electron irradiation showed excellent shape memory performance with strain fixity and strain recovery ratio of 95–99% [36]. Maimaitiming et al. [37] reported triple shape memory behavior for irradiation crosslinked polyolefin thermoplastic elastomers/polypropylene blends TPE/PP, shape fixity of 82 and 97%, and shape recovery of 88 and 94% were observed. Thermo-responsive shape memory polymer blends were investigated based on ethylene-1-octene thermoplastic elastomer (Engage 8440) and ethylene-propylene-diene EPDM rubber [30]. It was found that even without crosslinking, EPDM/ethylene-1-octene blends showed shape memory behavior due to good compatibility between both polymers. Le et al. [39] reported that it was possible to enhance the shape memory behavior of ethylene-1-octene (Engage 8200)/ethylene propylene diene elastomer EPDM blend by incorporating carbon black and crosslinking process. 

Economic reasons generate a need for the industry to manufacture new materials with additional features. Further strong emphasis is placed on environmental factors requiring the reuse of post-production waste and recycling of used products. It has become more and more desired to undertake research aimed at obtaining products containing recycled components. Responsible manufacturers try to maximize the use of the generated post-production waste and find new application areas for recycled products. Our investigations confirm that it is possible to obtain shape memory materials based on the waste EPDM rubber and ethylene-1-octene thermoplastic elastomers (TPE). In order to study the shape memory effect, three thermoplastic elastomers, Engage 8411, 8452, and 8180, together with EPDM rubber shred were used to prepare the final materials. The simple mixing method of blend preparation could be an economic advantage for the implementation of this approach in the industry. The viscoelastic properties of various TPE/recycled EPDM rubber blends were deeply analyzed. The correlation between the composition of the blend, its morphology, mechanical properties, and shape memory behavior is discussed. Overall, this work demonstrates that functional polymer materials could be prepared based on waste rubber. Therefore, we believe this approach opens new perspectives for the commercial application of rubbery waste and gives a base for further studies on the “smart” shape memory recycled materials.

## 2. Materials and Methods 

### 2.1. Materials

Waste rubber material obtained from the production of insulation materials was used in the form of EPDM carbon black filled insulation foam. The foam was firstly cleaned and cut into thin strips. Next, the strips were ground using rotational mixer Brabender Plasti Corder (Brabender GmbH, Duisburg, Germany) The device parameters were the following: temperature T = 25 °C, mixing time 15 min, speed of rotors 50 r⋅min^−1^. The rubber shred is further denoted as RS.

Three ENGAGE™ POE Ethylene Octene Grades, Dow Corning materials, among other commercially available grades of ethylene-1-octene thermoplastic elastomers, were selected as a binder for rubber shred due to their higher total crystallinity (factor influencing on the shape memory behavior and the mechanical properties) and various viscosity in a molten state (factor influencing on the mixing with the rubber shred).

- Engage 8180: melt index MI = 0.5 g⋅10 min^−1^, Mooney viscosity MV = 4, total crystallinity χ = 13%, durometer hardness (Shore A) 63.
- Engage 8411: melt index MI = 18 g⋅10 min^−1^, Mooney viscosity MV = 3, total crystallinity χ = 24%, durometer hardness (Shore A) 81.
- Engage 8452: melt index MI = 3 g⋅10 min^−1^, Mooney viscosity MV = 11, total crystallinity χ = 20%, durometer hardness (Shore A) 74.

### 2.2. Preparation of Rubber Shred/Thermoplastic Elastomer TPE Blends, Vulcanization of Mixtures

The RS/TPE blends were prepared using a Brabender micromixer. The main parameters of the device during blends preparation were as follows: temperature T = 100 °C, mixing time 10 min, speed = 50 r⋅min^−1^. 

The formulation of RS/TPE blend was: 100 gram of rubber shred (RS) per 100 grams of thermoplastic elastomer; Engage 8180, 8411, 8452, respectively. The blends are further denoted as RS/8180, RS/8411, RS/8452. 

Additionally, a dicumyl peroxide crosslinker (DCP) was added to the formulations in a proportion of 2 g DCP per 100 grams of RS/TPE mixture using a laboratory rolling mill. The main parameters of the device were the following: roll length L = 450 mm, roll diameter d = 200 mm, a rotational speed of the front roll r = 16 r⋅min^−1^, average roll temperature about 40 °C, preparation time for one mixture about 10 min. Blends modified by the addition of DCP are further denoted as RS/8180/DCP, RS/8411/DCP, RS/8452/DCP. 

Vulcanization of mixtures was carried out on the PH-ZPW90 hydraulic press (ZUP “Nysa” company, Nysa, Poland). A steel mold was placed between the two shelves of ironing. Device parameters were as follows: temperature T = 160 °C, vulcanization time according to the determined optimal vulcanization time τ_90_, pressure p = 200 bar.

### 2.3. Analysis of Shred Diameter

Sieve analysis of rubber shred RS was carried out according to the PN-ESO 3310-1: 2000 standard. The sieving analysis was done using an AS 200 device (Retsch GmbH, Düsseldorf, Germany) with a set of sieves with various mesh sizes. After mechanical shaking, fractions remained on the given sieve. The size distribution of fine grains was determined.

### 2.4. ATR-FTIR Analysis

Attenuated total reflection Fourier transport infrared spectra (ATR-FTIR) were recorded using a Thermo Fisher Scientific (Waltham, MA, USA) Nicolet 6700 FT-IR spectrometer equipped with a diamond ATR tool. The measurements were carried out at room temperature, the test conditions: a resolution of 4 cm^−1^, a 64-scan signal from 600 to 4000 cm^−1^ in absorbance mode.

### 2.5. Mechanical Properties at Static Conditions and Hardness of the Samples

The stress-strain curves and the values of elongation at break (E_b_), the stress at 100% tension (SE_100_), and tensile strength (TS) were measured at 25 °C using Zwick 1435 (ZwickRoell GmbH, Ulm, Germany) universal tensile machine, the crosshead speed of 500 mm⋅min^−1^ was used during tests. The six different dumb-bell shape specimens were prepared according to PN-EN ISO-37-2005 standard for each blend and tested. The reported parameters are the arithmetic mean, and standard deviation was calculated for six test results obtained for every formulation. Hardness in the Shore A scale was measured for cylinder shape samples (diameter 50 mm, thickness 10 mm) prepared according to PN-EN ISO 868:2005 standard. Ten tests were performed for each formulation. The reported data are the arithmetic mean and standard deviation calculated for the measured ten values of Shore A for each formulation.

### 2.6. Curing Characteristics of TPE/Rubber Shred Mixtures

Curing of RS/TPE/DCP mixtures was tested at the fixed angle of 0.5° and frequency of 1.67 Hz according to ASTM D5289. The curing plots were measured at 160 °C and the parameters such as the scorch time (t_Δ2_), the optimum cure time (τ_90_), cure rate (CRI), maximum elastic torque (*S′_max_*), minimum elastic torque (*S′_min_*), and increment of the elastic torque during curing Δ*S*′ defined as:

(1) $$\Delta S^{\prime }=S_{max}^{\prime }-S_{min}^{\prime }$$

were determined from the curing curves. 

### 2.7. Viscoelastic Properties, Damping Properties, Relaxation Behavior

Dynamic rheological measurements of uncured and cured RS/TPE and RS/TPE/DCP samples were performed using an oscillation rotational rheometer Ares G2 (TA Instruments). The measurements were done under shear using the plate-plate geometry (two parallel plates with a diameter of 25 mm). The storage shear modulus G′, and the loss shear modulus G″ were registered at temperatures of −20 °C, 0 °C and 25 °C as a function of oscillation strain in the range of 0.01–100%, the damping properties and the changes in the value of the mechanical loss tangent δ were analyzed. The oscillation sweep tests were carried out at an angular frequency of 10 rad⋅s^−1^. The measured values of the storage shear modulus G′ and loss tan δ at various temperatures during oscillation amplitude sweep tests from the oscillation strain range of 0.0015–0.1% were used to calculate the arithmetic mean values denoted as G′_LVR_ and loss tan δ_LVR_. The arithmetic mean was calculated using 20 values for each parameter.

Additionally, the rheological frequency sweep tests at 25 °C, in which the frequency was changed in the range of 0.1–628 rad⋅s^−1^ at the constant oscillation strain of 0.1% (linear viscoelastic region) were carried out. Stress relaxation tests were carried out under shear at 25 °C, the constant strain of 0.05% was applied, the relaxation curves were recorded during 100 s.

### 2.8. Shape Memory Effect

The cyclic shape memory test of thermoplastic elastomers, non-crosslinked and crosslinked blends was carried out. In the course of the measurement, a rectangular-shaped sample with an initial length *l*_0_ was heated in a drying oven at 100 °C, then the sample length was extended in the probe by 50% of its original length to its length *l_e_* and continued to be heated at 100 °C. Thereafter, the elongated sample was cooled in cold water to fix the length of *l_e_*. Upon cooling, the load was released, and the sample was observed to shrink from length *l_e_* to length *l_f_*. Reheating the sample (without load) at 100 °C caused the sample to return to its original length, from *l_f_* to *l_r_*. Two basic factors describing the shape memory behavior, such as shape fixity (F) and shape recovery ratio (RR) were calculated according to the following Equations (2) and (3) [30].

(2) $$F\;\left(\%\right)=\frac{l_{f}-l_{0}}{l_{e}-l_{0}}\;\cdot \;100$$

(3) $$\mathrm{RR}\;\left(\%\right)=\frac{l_{f}-l_{r}}{l_{f}-l_{0}}\;\cdot \;100$$

where F is shape fixity; RR is shape recovery, *l*_0_ is length of the sample measured at 25 °C before deformation of the sample, *l_e_* is length of the deformed sample measured at T > T_trans_, *l_f_* is length of deformed sample after shape fixation and unloading at T < T_trans_, *l_r_* is length of sample after shape recovery at T > T_trans_.

The parameter (F) indicates the ability to fix the programmed shape. The parameter (RR) indicates the ability of the sample to return to its original shape. Both parameters should achieve the value (F), and (RR) = 100% for an ideal shape memory material.

### 2.9. Morphological Properties

The morphological properties were analyzed using an optical microscope Carl Zeiss connected to a computer. Digital imaging Zen software was used to analyze the microscope images. 

## 3. Results

### 3.1. Characterization of Waste Insulation Foam

**Figure 1:**
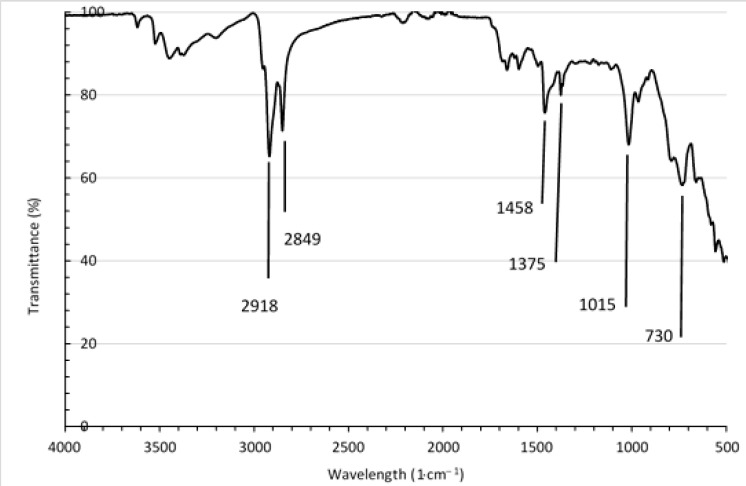
ATR-FTIR spectrum of waste rubber foam.

Figure 1 shows the ATR-FTIR infrared spectra of the foam waste material.

The spectrum of waste foams includes all groups characteristic for ethylene-propylene-diene EPDM rubber [40,41], such as broad overlapped peaks with a maximum at the wavenumbers of 2918 cm^−1^ and 2849 cm^−1^. The peaks at the wavelength of 2849 cm^−1^, 1375 cm^−1,^ and 965 cm^−1^ correspond to the valence vibration, deformation vibration, and symmetrical bending vibrations of the -CH group, respectively. The marked peaks at the wavelength of 2919 cm^−1^ and 1460 cm^−1^ are associated with the asymmetric stretch vibration and asymmetric deformation vibration of -CH_3_ group. The band at the wavenumber of 1458 cm^−1^.corresponded to the shear deformation vibrations of the -CH_2_- groups, while the broad peak in the region between 791 and 729 cm^−1^ to the skeletal vibration of the CH_2_ group. Additionally, the spectra show a peak at a wavelength of 659 cm^−1^ that can be attributed to valence stretch vibration of C-Cl groups derived from the additives regulating the flammability of the mixture (flame retardants). A broad peak at a wavelength of about 3447 cm^−1^, attributed to stretching vibration of the OH groups, was also observed. This peak resulted from the presence of the antiaging and anti-oxidative additives containing -OH groups. 

**Figure 2:**
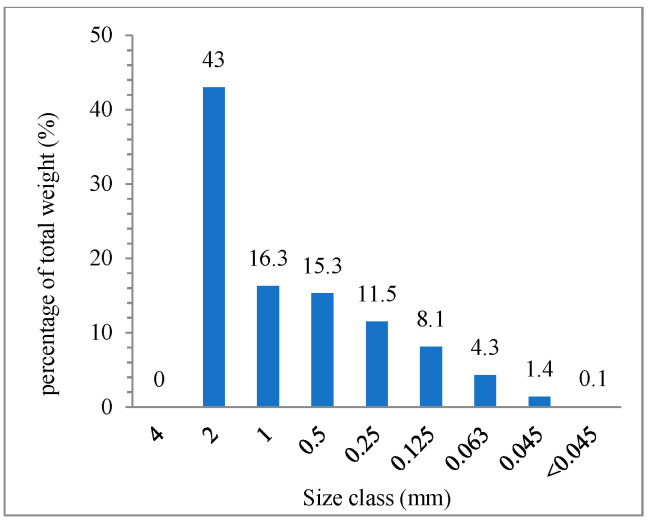
Percentage of shred weight on screens with decreasing mesh size.

A reduction in shred diameter was observed after grinding on a Brabender Mixer for 15 min. The largest grain diameter was less than 4 mm. The sieve analysis (Figure 2) showed that the grounded material’s largest fraction (43% by weight) was the grains with a diameter bigger than 2 mm. The weight percentage of the grains in diameter between 2 mm and 1 mm was 16.3%. The total weight percentage of the rubber shred with a diameter lower than 1 mm was 40.7%. 

### 3.2. Curing Characteristic of the Blends

**Figure 3:**
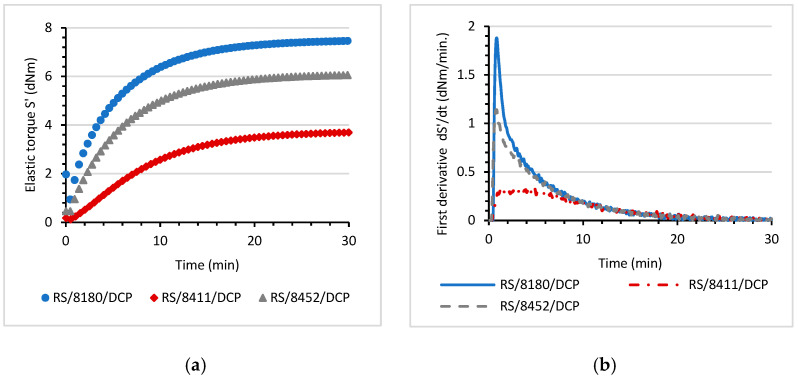
(**a**) The elastic torque S′ (dNm) as a function of time, (**b**) the first derivative of elastic torque dS′/dt (dNm⋅min^−1^) as a function of time; each for various RS/TPE/DCP blends.

The curing process of the prepared RS/8180/DCP, RS/8411/DCP, RS/8452/DCP blends were analyzed and the change in the value of elastic torque (S′) as a function of time was determined (Figure 3a).

All investigated RS/TPE/DCP blends showed typical curing plots characteristic for elastomers cured by peroxide. Although, the type of thermoplastic elastomer used to prepare the blend strongly influenced the kinetics of curing the final RS/TPE/DCP material. The RS/8180/DCP blend showed the highest increase of elastic torque (S′) during curing. In contrast, the progress of the peroxide curing reaction of RS/8452/DCP blend was restricted, resulting in lower values of S′ after 30 min of curing. The first derivative dS′/dt versus curing time was determined (Figure 3b). This parameter provides information about the curing speed and about the ultimate state of curing. For the RS/TPE/DCP blends, the local maxima of the first derivative dS′/dt were observed at different times, indicating that thermoplastic elastomer properties affected the curing speed. Particularly for RS/8452/DCP blend, a slower speed of curing was recorded. Moreover, the maximum value of the first derivative dS′/dt was significantly lower, confirming that the achieved ultimate state of cure was lower. With respect to the reaction speed, both 8180 and 8411 significantly affected the cure rate of the RS/TPE/DCP blends. The maximum cure rate was recorded at a shorter time compared to the RS/8452/DCP blend. Although RS/8180/DCP and RS/8411/DCP differed in the maximum value of the first derivative dS′/dt, the higher ultimate state of cure was observed for RS/8180/DCP blend. 

Table 1 compiles the characteristic parameters of curing at 160 °C. The lowest value of the minimum of elastic torque (*S′_min_*) was observed for RS/8411/DCP, indicating the lowest viscosity of plasticized material. It is in accordance with the values of Mooney viscosity given by the producer. Among the TPEs used to prepare RS/TPE/DCP blends, the 8411 Engage has the lowest Mooney viscosity. The type of TPE influenced the incubation time (t_i_,). After that, the curing started. The longer incubation time (t_i_) observed for the RS/8180/DCP blend resulted from, the higher viscosity. The diffusion of formed free radical was restricted due to the higher viscosity of the 8180 TPE sample, and curing started after a longer time. For blends containing the 8180 and 8411 TPEs a slightly longer scorch time (t_Δ__5_) was determined. The optimal cure time (τ_90_) for RS/8411/DCP was longer, and that can be an important factor from a practical point of view because a longer cure time basically means higher costs of production. The increment of elastic modulus during curing (Δ*S*′) and cure rate index (CRI) parameters, related to the increase of elastomer crosslink density, were analyzed. A higher value of both parameters indicates that more cured material is formed. It should be underlined that type of thermoplastic elastomer used to prepare RS/TPE/DCP blend strongly influenced the formation of a 3D network of cured material and the values of CRI and Δ*S*′ parameters. A further increase of elastic modulus during curing (Δ*S*′) was detected for RS/8180/DCP and RS/8452/DCP, confirming that peroxide curing occurred with enhanced activity as compared with the RS/8411/DCP blend. 

### 3.3. Mechanical Properties and Hardness of the Material

The incorporation of the shredded recycled rubber into elastomer or polymer blends can lead to the deterioration of the mechanical properties [9]. That is the main disadvantage of material recycling. It is crucial to investigate the mechanical properties of the composite material containing rubber shreds. The mechanical properties of recycled material should not be worse than the original (not recycled) material. The deterioration of the strength of the material could be balanced by the additional curing of the prepared blends [9,10,11,12].

Table 2 presents the results of static mechanical tests during stretching, together with the standard deviation for the neat TPE and their blends.

Thermoplastic elastomers are polymers with strong permanent deformation in comparison with conventional vulcanized rubbers. For neat TPE 8411 and 8452, the elongation at break E_b_ was more than 1100%. TPE 8180 showed even larger elongation, more than 1600%. The incorporation of rubber shred reduced both the tensile strength (TS) and the elongation at break (E_b_) of all RS/TPE blends. Peroxide curing, as expected, enhanced static mechanical properties to the acceptable level for commercial rubber products’ applications. The stress at 100% of elongation (SE_100_) and the TS increased meaningfully. Additionally, after curing, the further reduction in elongation at break (E_b_) was observed. For RS/8452/DCP and RS/8180/DCP blends, vulcanization caused an approx. A 2-fold decrease in elongation value at break, E_b,_ and a 4-fold increase in the value of tensile strength, TS compared with uncured RS/8452 and RS/8180 blends. In the case of RS/8411/DCP no significant difference in elongation values at the break after curing was observed. The difference in the degree of crystallinity between 8411, 8452, and 8180 TPE was the main factor influencing the values of stress at 100% elongation (SE_100_) for RS/TPE blends. After curing, the differences in SE_100_ were less visible.

The Shore hardness of the waste material and obtained blends was determined. The waste foam was outside the Shore A range, so performing a Shore 0 measurement for the waste was necessary. The Shore 0 hardness of the foam waste material was 28 ± 4. For the neat thermoplastic elastomer Engage 8180, the values of Shore A hardness were almost similar, and in the case of Engage 8411 and 8452, slightly lower than the values given by the producer. Probably the processing of the material influenced the structure of the neat 8411, 8452 and its crystallinity. The mixing of the neat TPE with rubber shred decreased the hardness of the blend. The hardness value reduction after the rubber shred was lower for the RS/8411 and RS/8452 blends. Both thermoplastic elastomers, 8411, 8452 have lower viscosity in the molten state as comparing with Engage 8180. This was the reason that they acted as a better binder as they were able to penetrate pores of the foam rubber shred during mixing at a higher temperature. Additionally, the ratio of crystallinity of 8411 and 8452 is higher than Engage 8180, and consequently, both materials, RS/8411 and RS/8452 were harder compared to RS/8180. The curing of the RS/TPE/DCP blends enhanced the hardness of the material as the crosslink density of the elastomer in the blend increased to the level acceptable for the rubber industry. 

The obtained results of mechanical properties differed slightly depending on the thermoplastic elastomer used to prepare the RS/TPE blend that indicated that all chosen TPEs could be used as a binder for the rubber shred. Moreover, the mechanical properties of prepared material containing recycled foam are acceptable, and the material can be used once again in the industry.

### 3.4. Viscoelastic Behavior and Damping Properties of Material at Ambient (25 °C) and Low (−20 °C) Temperature

#### 3.4.1. Oscillatory Measurements at Variable Strain Amplitude

The low mechanical strength and shape recovery stress are two main factors limiting shape memory polymers’ application [15]. The addition of high modulus inorganic or organic fillers and fibers can significantly improve the elastic modulus and recovery stress [13,15], leading to a higher shape recovery ratio. The rubber shred is a material that could act as an additive influencing the viscoelastic behavior, especially its elasticity, leading to better strain recovery after removing the external force. Thus, viscoelastic studies for the proposed materials are crucial for commercial viability. 

**Figure 4:**
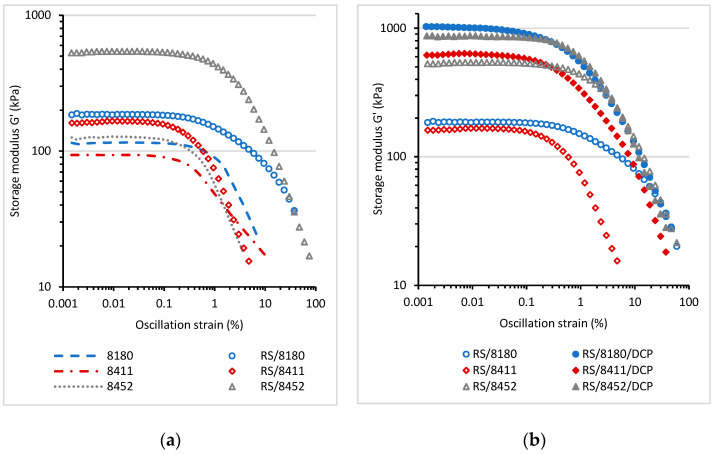
The storage shear modulus G′ as a function of oscillation strain (%) for neat TPE and RS/TPE blends (**a**); for cured RS/TPE/DCP blends (**b**). Condition of measurements: angular frequency 10 rad⋅s^−1^, temperature 25 °C.

**Figure 5:**
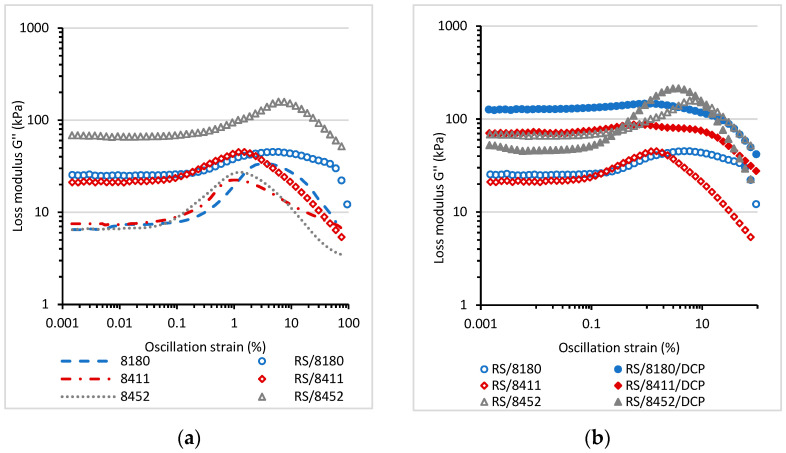
The loss shear modulus G″ as a function of oscillation strain (%) for neat TPE and RS/TPE blends (**a**) and cured RS/TPE/DCP blends (**b**). Conditions of measurements: angular frequency 10 rad⋅s^−1^, temperature 25 °C.

The rheological tests on prepared blends were carried out at a variable amplitude and constant temperatures of 25 °C (Figure 4 and Figure 5). Based on these measurements, it is possible to determine the linear viscoelastic range, in which the viscoelastic response of the material is constant and not affected by the applied oscillation strain (deformation). Figure 4a shows the changes in the storage shear modulus G′ values at 25 °C as a function of oscillation strain for the neat thermoplastic elastomers and for RS/TPE blends. 

The storage shear modulus (G′) of neat Engage thermoplastic elastomers was lower than G′ values determined for RS/TPE blends. Rubber shred is elastic, partially vulcanized material that, under dynamic deformation, can act as an additive that changes the viscoelastic behavior and enhances the values of G′ modulus. It should also be noted that the foam used to prepare rubber shred was filled with carbon black. Carbon black is an active filler able to reinforce rubber due to factors such as the hydrodynamic effect and the formation of filler-rubber interactions or the particle-particle interactions. The presence of carbon black was probably also a crucial factor causing the significant enhancement of G′ for all RS/TPE blends. The type of the thermoplastic elastomer TPE used to prepare RS/TPE blend also affected the increase of G′. The lower values of storage shear modulus were recorded for the rubber shred blend prepared using the 8411 thermoplastic elastomer. Moreover, the range of linear viscoelastic region for RS/8411 blend was shorter. The changes in the viscoelastic properties and the material’s structure due to applied deformation occurred at a lower level of deformation. The values of Mooney viscosity and melt flow index of TPE 8411 Engage, provided by producent, suggest that the molecular weight of this TPE is lower as compared with other TPEs used in this study, and it probably influenced the dynamic viscoelastic behavior of the RS/8411 blend. The curing of blends caused a further increase of the storage shear modulus G′ (Figure 4b) together with the extension of the linear viscoelastic range for all RS/TPE/DCP formulations.

Figure 5 displays the values of loss shear modulus (G″) measured for RS/TPE and RS/TPE/DCP blend as a function of oscillation strain at 25 °C. For the neat thermoplastic elastomers and RS/TPE a maximum in the value of loss modulus G″ was observed, indicating the maximum energy loss (dissipation) during deformation. After exceeding the maximum value, the loss of shear modulus G″ dropped drastically, showing the destruction of interphase interactions and the material structure. The incorporation of rubber shred into RS/TPE blend increased the energy dissipation, and higher values of G″ modulus was observed compared to neat thermoplastic elastomers. The curing of blends caused a further increase of the loss of shear modulus G″ for all RS/TPE/DCP compositions.

Insulation materials are usually applied in outdoor elements. Therefore, the viscoelastic dynamic properties at various, also minus temperatures, are important. The storage shear modulus G′ and loss shear modulus G″ as a function of oscillation strain at −20 °C and 0 °C were studied (Table 3; Supplementary Materials Figure S1: The storage shear G′ and the loss shear modulus G″ for neat thermoplastic elastomers, RS/TPE blends, and for cured blends RS/TPE/DCP. Conditions of measurements: angular frequency 10 rad⋅s^−1^, temperature −20 °C.; Figure S2: The storage shear G′ and the loss shear modulus G″ for neat thermoplastic elastomers, RS/TPE blends, and for cured blends RS/TPE/DCP. Conditions of measurements: angular frequency 10 rad⋅s^−1^, temperature 0 °C). As expected the values of storage shear modulus G′ and loss shear modulus G″ measured for the linear viscoelastic range at −20 °C and 0 °C increased in comparison to these observed at 25 °C. Linear viscoelastic ranges were comparable for all RS/TPE blends, although values at -20°C were lower when compared with values at 25 °C temperature or with neat thermoplastic elastomers (TPE). The RS/TPE blend application at −20 °C should be restricted to a lower level of dynamic deformation, less than 0.5%. A higher level of dynamic deformation caused changes in viscoelastic properties of RS/TPE blends, leading to the decrease of storage shear modulus G′ and loss shear modulus G″. After curing the linear viscoelastic range for RS/TPE/DCP blends was extended. 

From an industrial point of view, the damping properties of the final material at various temperatures are important. The parameter of such a reduction is the ratio between the loss modulus G″ and the storage modulus G′, tan δ, and it measures the damping properties of the material. The value of tan δ is the ratio of the energy lost to the energy retained during the cyclic deformation of the material, and it indicates the amount of hysteresis present during the deformation. This means that when tan δ is high, the material exhibits higher attenuation than that with lower values of tan δ. The values of tan δ measured at various temperatures for the linear viscoelastic region, together with the values of the storage modulus G′ are compiled in Table 3. The incorporation of recycled rubber shred increased the values of loss tan δ indicating stronger energy dissipation and better damping properties for all studied temperatures, 25 °C, 0 °C, −20 °C. Curing of the blends led to an increase of the storage modulus G′, resulting in lower values of tan δ for RS/TPE/DCP samples. Although tan δ values increased at lower temperatures, the changes were different for the varied thermoplastic elastomers used to prepare RS/TPE blends. For the blends based on Engage 8411 and 8452, a strong increase of tan δ at −20 °C was observed, identifying that the damping properties of the material changed drastically. On the contrary, it was possible to obtain materials that showed good dynamic properties after curing, even at 0 °C and −20 °C, if Engage 8180 was used; RS/8180/DCP.

#### 3.4.2. Oscillatory Measurements at Variable Angular Frequency at Ambient Temperature

**Figure 6:**
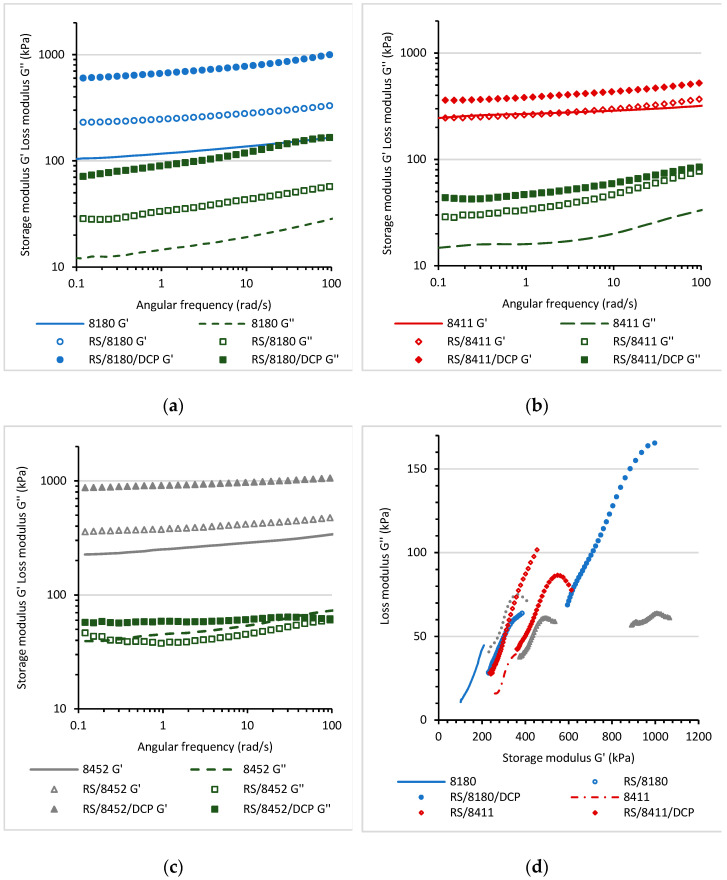
Dependence of the storage modulus and loss modulus of Engage 8180, RS/8180 and RS/8180/DCP blends (**a**); Engage 8411, RS/8411 and RS/8411/DCP blends (**b**); Engage 8452, RS/8452 and RS/8452/DCP blends (**c**) as a function of angular frequency and (**d**) Cole-Cole plot for the samples.

For all RS/TPE and RS/TPE/DCP blends, the storage shear modulus G′ and loss shear modulus G″ were registered at 25 °C as a function of angular frequency (ω) (Figure 6a–c). Additionally, to analyze the influence of the various thermoplastic elastomers on the viscoelastic behavior, the relationship between the storage G′ and loss modulus G″ was determined based on the Cole-Cole plot (Figure 6d). The Cole-Cole diagrams provide information about the relaxation processes occurring in multiphase materials. For a partially miscible blend, the Cole-Cole plot is an almost semicircular arc/curve. The more semicircular shape of the plot, the better compatibility of the blend [42]. For the semicircular Cole-Cole curves, the inverse of the maximum frequency corresponds to the horizontal tangent at the top of a circle [43]. The Cole-Cole diagrams for RS/TPE and RS/TPE/DCP blends showed immiscibility of the blends and a major deviation from semicircular shape. Therefore, the calculation of the average relaxation time of the phase was impossible. Nevertheless, it was possible to analyze the changes in phase miscibility for various thermoplastic elastomers used as a binder for a rubber shred. 

The storage shear modulus G′ and loss shear modulus G′ as a function of angular frequency for blends based on the thermoplastic elastomer Engage 8180 are shown in Figure 6a. The mixing of rubber shreds with 8180 TPE increased the values of storage shear modulus G′ and loss shear modulus G″. Partially vulcanized rubber shreds acted as a reinforcing additive, increasing the elasticity of the material and its strength. The curing of the material caused an additional increase of the G′ and G′ parameters but did not influence the compatibility of the blend. The Cole-Cole plot of the RS/8180/DCP blend did not have a semicircular shape compared to RS/8411/DCP and RS/8452, indicating worse miscibility of the material.

A slight increase of G′ was observed for RS/8411 at higher values of angular frequency (short relaxation time). At lower values of frequency (long relaxation time), the storage shear modulus G′ values were similar to the values observed for the neat Engage 8411. The curing of the RS/8411/DCP blend also resulted in a less significant increase of storage modulus G′ compared with RS/8452/DCP and RS/8180/DCP. Still, the Cole-Cole plots revealed that the blend’s compatibility was better compared to other RS/TPE/DCP blends. The lower viscosity of molten 8411 during mixing with rubber shred meant that the thermoplastic elastomer was more effectively mixed with rubber shred at the same shear rate of mixing. At the same time curing caused the formation of inter-phase bonds between thermoplastic elastomer and rubber shred, increasing their compatibility.

It is evident that with the preparation of blends of various ethylene-1-octene thermoplastic elastomers and insulation foam rubber shreds, it was possible to design newly recycled, more environmentally friendly materials with the optimal viscoelastic characteristic for the application in industry. 

### 3.5. Stress-Relaxation Behavior

**Figure 7:**
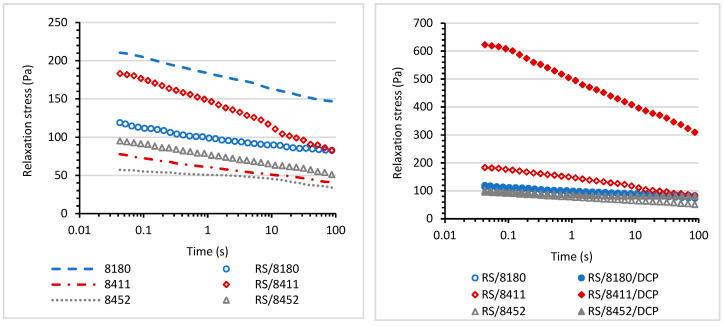
Relaxation stress as a function of the logarithm of time.

Relaxation tests were performed to estimate the influence of various TPEs on the behavior of blends under constant shear deformation. Various processes occur in deformed polymeric material under constant strain: the rearrangement and motion of the elastomer chains, microdomains rupture, and breaking of crosslinks. The relaxation phenomenon of the blends is strongly affected by the structure of the material, types of interfaces, and their strength. As a result, stress relaxation may occur due to one-stage or multi-stage mechanisms [42]. The stress decay of blends containing recycled rubber shred was monitored at a constant shear strain of 0.5% over 100 seconds. Figure 7 illustrates plots between the stress and the logarithm of time for the blends.

The applied stress during relaxation measurements results in relaxation stress. It can be dissipated or stored in deformed chains that are not able to retract. Thus, the crosslinking prevents the rearrangement of the chains and can restrict the dissipation of the energy during deformation. The homogeneity and the compatibility of the interphase morphology can also influence the dissipation of the energy. The RS/TPE and RS/TPE/DCP blends showed typical relaxation behavior, initially fast stress decay that slowed down with time (Figure 7). Two straight lines of unequal slops were observed for stress relaxation plots of RS/TPE and RS/TPE/DCP blends, indicating various relaxation mechanisms at shorter and longer times. The highest initial stress was observed for the RS/8411/DCP blend indicating the higher energy stored in the deformed structure. Moreover, this blend also showed faster stress decay. The decay of stress at various times of relaxation may occur due to the reorientation of small segments or domains of both components of the blends. In contrast, the further stage of relaxation may be related to a long-range rearrangement of molecular chains at the interphase between components of the blend, or the failure of bonding at the interphase. 

**Figure 8:**
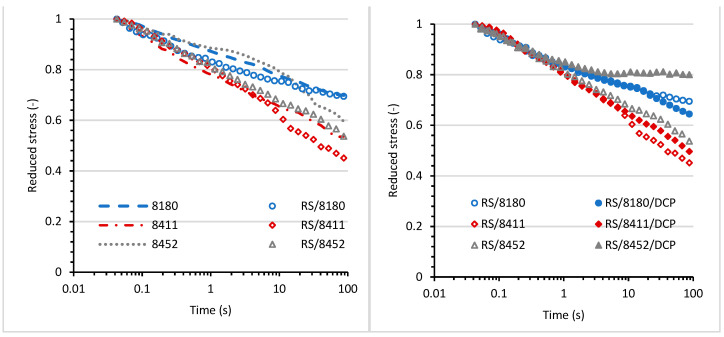
Reduced relaxation stress as a function of time.

The relaxation plots were normalized with respect to the initial stress to estimate the differences between the degrees of relaxation of the RS/TPE blends. The normalized curves are presented in Figure 8. The type of thermoplastic elastomer used to prepare the RS/TPE blend has a meaningful influence on the degree of relaxation. At the shortest time of relaxation, up to one second, the stress decay was similar for all blends, even cured ones, but the differences in stress decay for various blends were visible at longer times. A stronger reduction in relaxation stress was observed for rubber shred blends based on 8452 and 8411 thermoplastic elastomers as compared with RS/8180 blend. As expected, curing both RS/8411 and RS/8452 blends led to a slower relaxation speed and lower stress decay during the same period of time. Curing and its influence on the relaxation speed were clearly visible for the RS/8452 blend. Probably after curing, a higher number of crosslinks was formed in the 3D network, thereby restricting the mobility of polymeric chains compared with the RS/8452/DCP blend. Curing did not significantly influence the relaxation behavior of the RS blends based on the 8180 thermoplastic elastomer.

### 3.6. Morphology of the Blends

**Figure 9:**
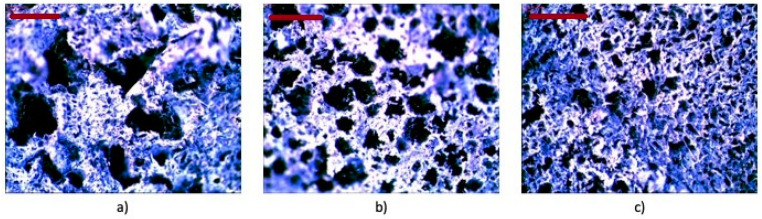
The microscopic images, after approximately 300× magnification of the RS/8452 (**a**); RS/8180 (**b**), RS/8411 (**c**).

The factors such as the morphology of the phase-separated systems, the distribution and diameters of dispersed domains, interfacial adhesion and strength of interfacial interactions, or the presence of the chemical bonds between two blend phases were reported to influence the viscoelastic behavior and mechanical properties of multiphase shape memory materials, as well as the shape memory behavior [14]. The microstructure of prepared blends was studied using optical microscopy. The waste rubber foam used to prepare the rubber shred was a porous material, with the average diameter of the pores 0.49 ± 0.11 mm. All blends showed two phases of immiscible morphology. No air bubbles were observed in blends with thermoplastics; probably, domains of one material were embedded in another material. As the type of thermoplastic elastomer TPE was changed, the morphology of the resulting systems clearly differed (Figure 9). In the blends, the continuous phase was observed to be derived from the thermoplastic, in which fragments of fine dust of various diameters were dispersed. The diameters of the dispersed rubber shred phases were in the range of 0.47–0.30 mm for RS/8452 blend, 0.31–0.13 mm for RS/8180 blend, and 0.24–0.07 mm for RS/8411 blend. The morphology analysis was in accordance with the observations from viscoelastic studies. The smaller diameters of domains observed for RS/8411 blend indicated higher compatibility of the material. The analysis of Cole-Cole plots also confirmed it; the more semicircular shape of the curve was observed. The largest domains diameters were measured for RS/8452 blend. For the RS/8452 blend, the strongest enhancement of the storage shear modulus G′ at 25 °C as a function of oscillation strain was reported. The larger domains of partially vulcanized EPDM rubber increased more strongly the elastic response of the material under deformation leading to higher values of storage shear modulus G′.

### 3.7. Shape Memory Effect

**Figure 10:**
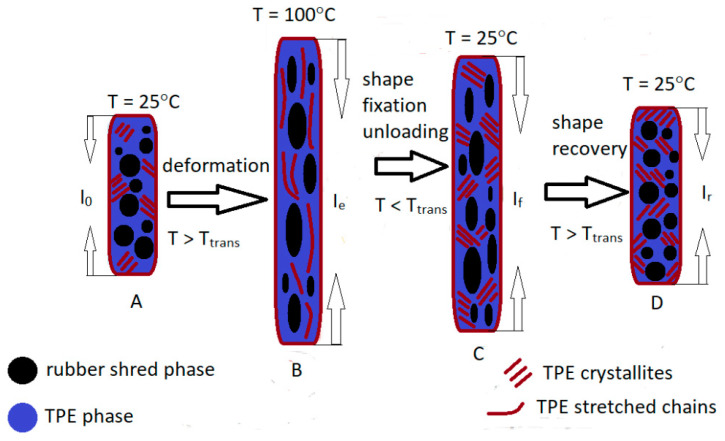
Schematic representation of sample deformation during shape memory tests.

The shape memory effect was studied while stretching the sample according to the scheme shown in Figure 10. At room temperature (A) in the RS/TPE blends, the rubber phase is dispersed in the TPE phase that contains amorphous and crystalline areas. During the stretching of the blend mixtures at the temperature of 100 °C, chains of both soft (rubber dust) and hard (TPE) phases orient themselves in the direction of stretching (B). After cooling down to room temperature, the TPE crystallizes, limiting the mixtures’ relaxation after the stress is released and stabilizes the programed shape (C). Upon reheating without stress (crystallite melting), the chains loosen again and return to their original undistorted shape, and upon cooling, they crystallize again (D). The higher the content of the crystalline phase in the sample, the better the stability of both the original and the desired (predetermined) shape. Therefore, the higher the content of the crystalline phase, the better the shape fixity (F) will be. The shape fixity factor F indicates the durability of the given shape of the material. The higher it is, the more durable the shape given to the material and the most like the original pattern.

**Figure 11:**
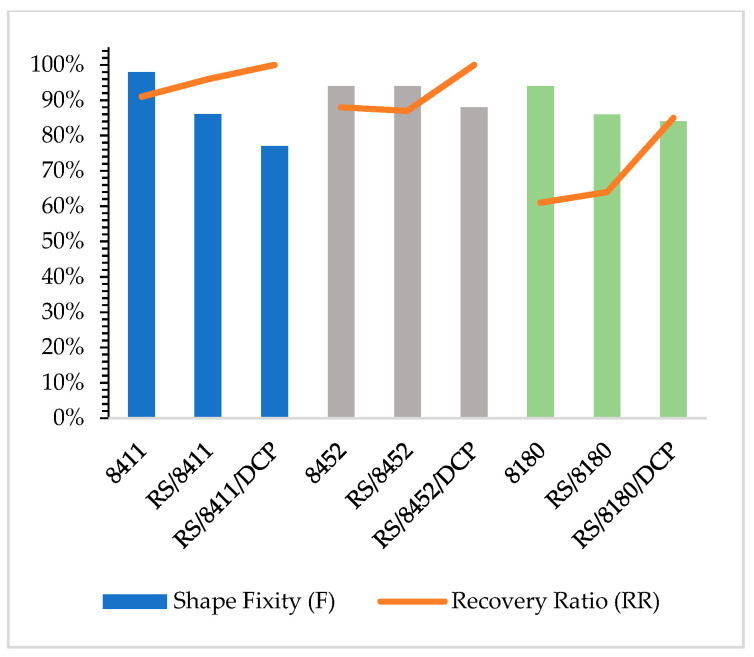
The shape fixity F factor and the recovery ratio RR parameter for neat thermoplastic elastomers Engage 8411, 8452, 8180, RS/TPE and RS/TPE/DCP blends.

The shape fixity results are shown in Figure 11 (columns) and Table S1 (Supplementary Materials, Table S1: The shape fixity F and the recovery ratio RR calculated for the TPE, RS/TPE and RS/TPE/DCP blends).

The incorporation of rubber shred decreased the shape fixity factor F as compared with neat thermoplastic elastomers. For RS/TPE blends, the crystallization/melting transition of TPE phase is responsible for fixing the temporary shape. The presence of the second phase rubber shred (RS) may influence the crystallization of the blend during cooling. A lower degree of crystallinity of the thermoplastic elastomer phase in the RS/TPE material was probably responsible for the decrease of the shape fixity compared to the neat TPE. Moreover, for the RS/8452 blend, the highest value of the shape fixity F (94%) was observed as compared with RS/8411 (86%) and RS/8180 (86%). The various morphology of the RS/8452 blend and the presence of large areas of 8452 TPE phase without the dispersed rubber shred could be responsible for this effect. Probably for the RS/8452 blend, the crystallization of 8452 phase occurred faster during cooling due to higher mobility of TPE chains, not restricted by the presence of the elastic RS domains and interphase interactions. Opposite, more homogenous morphology, smaller diameters of the dispersed RS phase generated larger interphase contact influencing the crystallization of the RS/8180 and RS/8411 during cooling, resulting in a stronger decrease of shape fixity (F) as compared with neat 8180 and 8411 TPE. 

For all RS/TPE blends the recovery ratio (RR) parameters were higher than this observed for neat thermoplastic elastomers, indicating better shape memory behavior. The values of RR for RS/TPE were respectively: RS/8452 (87%), RS/8411 (96%), RS/8180 (64%). According to the literature [15], two main factors influencing shape memory behavior, especially shape recovery, are low mechanical strength and shape recovery stress. To improve the shape recovery ratio, usually inorganic or organic fillers with high modulus are added to shape memory materials [13,15]. As we showed, analyzing the viscoelastic properties of prepared blends, rubber shred is elastic, partially vulcanized material that under dynamic deformation can change the viscoelastic behavior and improve the values of G′ modulus leading to better strain recovery after removing the external force. Moreover, the incorporated as an additive to the blend rubber shred was filled by carbon black. Carbon fillers were found to be reinforcing fillers able to enhance the shape recovery stress of shape memory polymers [13]. The above-mentioned factors affected the shape recovery of the RS/TPE blends.

After crosslinking, a further decrease of shape fixity F factor was observed; however, the shape fixity level remained in an acceptable range from 77% (RS/8411/DCP) to 88% (RS/8452/DCP). The curing process led to a decrease of flexibility that resulted in lower shape fixity F values and a higher recovery ratio (RR), in range from 85% (RS/8180/DCP) to 100% (RS/ RS/8452/DCP). 

Summarizing the shape recovery trend, the thermoplastic elastomer 8411, with the highest crystalline phase percentage (24%), has the highest percentage of shape recovery, while sampling 8180 (16% of crystalline phase) is the lowest one. The addition of rubber shreds, and above all, the crosslinking of the blends, resulted in a significant enhancement of the shape recovery ratio.

**Figure 12:**
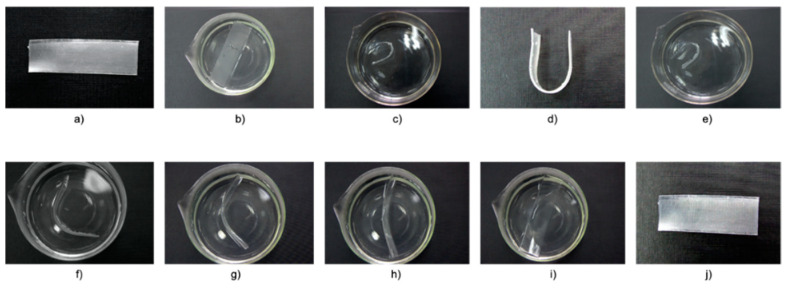
A series of photos demonstrating the shape memory behavior of the thermoplastic elastomers Engage 8411: (**a**) Initial sample; (**b**) softening the sample in hot water; (**c**) fixation of a temporary shape by the emergence in cold water; (**d**) fixed temporary shape; (**e**) immersing the temporary sample in hot water to restore its initial shape; (**f**–**i**) recovering of the original sample shape at various stages; (**j**) sample after final recovery.

**Figure 13:**
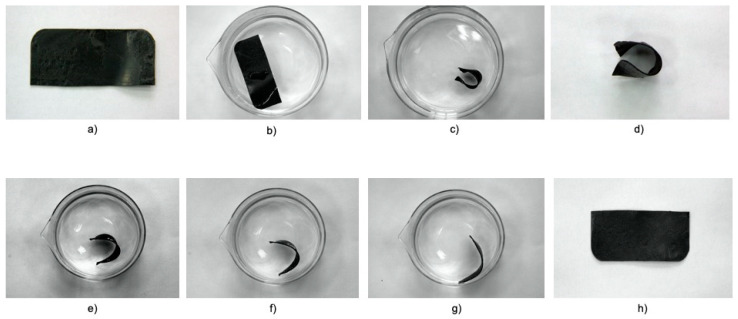
A series of photos demonstrating the shape memory behavior of RS/8411/DCP crosslinked blend: (**a**) Initial sample; (**b**) softening of the sample in hot water; (**c**) fixation of a temporary shape by the emergence in cold water; (**d**) fixed temporary shape; (**e**) immersing the temporary sample in hot water to restore its initial shape; (**f**,**g**) recovering of the sample shape at various stages; (**h**) sample after final recovery.

The shape memory test was also carried out in hot water (Figure 12 and Figure 13).

The rectangular-shaped sample was placed in a beaker with hot water. Then, after taking it out, it was temporarily shaped and fixed by immersion in cold water. After the temporary shape had been hardened, the sample was immersed in hot water again to regain its original shape. The series of photos for both neat thermoplastic elastomer Engage 8411 (Figure 12) and RS/8411/DCP blend (Figure 13) is additional confirmation of the shape memory behavior. A new “smart” material can be created by mixing waste rubber and various thermoplastic elastomer. This procedure can be a great advantage considering the utility of the demonstrated application in industry.

## 4. Conclusions

The application of thermoplastic elastomers (TPEs) of the ethylene-1-octene type as components of blends generates the possibility of using rubber dust, or production waste, to obtain new products. From the point of view of the economics of production, it is a significant advantage. By using ethylene-1-octene with varied basic properties, the behavior of blends containing the EPDM rubber shred (RS) can be designed. The ground EPDM waste product was mixed using a typical rubber industry equipment mixer.

The properties of ethylene-1-octene used, such as melt viscosity and the degree of crystallinity, were the main factors influencing the phase morphology of the resulting RS/TPE blends, their viscoelastic properties, and the observed shape memory effect.

The introduction of the waste EPDM rubber shred into the TPE matrix was associated with deterioration of static mechanical properties, tensile strength, and hardness. However, by crosslinking the RS/TPE mixtures, it was possible to obtain a material with improved mechanical properties compared to neat TPE, higher tensile strength, comparable hardness. It was observed that the type of TPE used influenced the crosslinking kinetics of the RS/TPE system. The highest increase of elastic torque (S′) and cure rate index (CRI) was recorded for the blend based on Engage 8180. No significant influence of the type of TPE used on the optimal cure time (τ_90_) was observed. All RS/TPE blends achieved the elastic torque S′ plateau in less than 20 min at 160 °C.

The incorporation of EPDM rubber shreds enhanced the dynamic mechanical properties of RS/TPE blends. A significant influence of the thermoplastic elastomer properties on the viscoelastic behavior of RS/TPE and RS/TPE/DCP blends, especially the storage shear modulus G′ values was observed. That effect resulted from various morphology and formed blend structures determining the strength of the interfacial interactions. Crosslinking caused further increase of storage shear modulus G′, and loss shear modulus G″. The presence of rubber shred caused stronger energy dissipation leading to higher values of tan δ as compared with neat thermoplastic elastomers. The values of tan δ decreased after curing.

Overall, it can be concluded that ethylene-1-octene copolymers can be used as binders for EPDM rubber shred due to their high compatibility with recycled rubber. The mechanical and viscoelastic properties of prepared materials containing recycled foam are acceptable. Such materials can be used again in industry. From the industrial point of view, it is important that all RS/TPE blends show the shape memory effect. Among RS/TPE blends, the highest value of shape fixity F and shape recovery was recorded for RS/8452 blends indicating that the enhanced mechanical and viscoelastic properties could be a crucial factor influencing on the shape memory effect. Compared with other RS/TPE blends, the higher storage modulus and tensile strength of the material, as compared with other RS/TPE blends, led to better stabilization of programmed shape during stretching. On the other hand, the higher relaxation speed reported for RS/8452 and RS/8452/DCP blends allowed faster and better shape recovery.

## Figures and Tables

**Table 1:** Curing characteristic at 160 °C. The minimum elastic torque *S′_min_*, the increase of elastic modulus during curing Δ*S*′, cure rate index CRI, incubation time t_i_, the scorch time τ_Δ5_, optimal curing time τ_90_.

	*S′_min_* dNm	Δ*S*′ * dNm	CRI dNm⋅min^−1^	t_i_ s	τ_Δ5_ s	τ_90_ min
RS/8180/DCP	0.87	6.59	1.88	39	69	13
RS/8411/DCP	0.10	3.60	0.34	19	70	17
RS/8452/DCP	0.31	5.75	1.14	17	37	14

* Δ*S*′ = *S′_max_* − *S′_min_*

**Table 2:** The static mechanical properties of TPEs and their blends. Stress at 100% of elongation SE_100_, tensile strength TS, elongation at break E_b_, hardness (Shore A).

	SE_100_ MPa	TS MPa	E_b_ %	Shore A
8180	1.22 ± 0.04	6.79 ± 1.63	1697 ± 316	67.4 ± 3.6
RS/8180	0.96 ± 0.04	1.80 ± 0.22	523 ± 88	36 ± 4.0
RS/8180/DCP	4.19 ± 0.20	8.09 ± 0.27	190 ± 5	66 ± 2.0
8411	3.55 ± 0.06	6.16 ± 1.05	1182 ± 205	72 ± 2.0
RS/8411	1.10 ± 0.22	1.16 ± 0.25	140 ± 48	49 ± 1.0
RS/8411/DCP	4.53 ± 0.25	7.00 ± 0.35	189 ± 9	70 ± 2
8452	2.98 ± 0.08	6.50 ± 0.54	1118 ± 74	68.2 ± 1.8
RS/8452	1.27 ± 0.17	2.01 ± 0.38	424 ± 63	53 ± 5
RS/8452/DCP	4.23 ± 0.20	8.09 ± 0.24	188 ± 5	78

**Table 3:** The storage shear modulus G′ and loss tan δ measured for the linear viscoelastic region at various temperatures.

	25 °C	25 °C	0 °C	0 °C	−20 °C	−20 °C
Sample	G′_LVR_ (kPa)	tan δ_LVR_ (−)	G′_LVR_ (kPa)	tan δ_LVR_ (−)	G′_LVR_ (kPa)	tan δ_LVR_ (−)
8180	114.7 ± 0.8	0.062 ± 0.003	518.1 ± 9.2	0.085 ± 0.004	847.3 ± 12.2	0.208 ± 0.005
RS/8180	185.3 ± 0.9	0.136 ± 0.002	558.0 ± 11.9	0.193 ± 0.007	890.0 ± 22.5	0.269 ± 0.004
RS/8180/DCP	992.4 ± 27.8	0.129 ± 0.005	1142.6 ± 26.7	0.195 ± 0.006	1602.4 ± 68.9	0.193 ± 0.009
8411	93.05 ± 0.7	0.083 ± 0.004	436.9 ± 7.1	0.156 ± 0.003	669.4 ± 8.7	0.193 ± 0.004
RS/8411	163.4 ± 2.2	0.133 ± 0.004	680.7 ± 6.9	0.270 ± 0.008	991.9 ± 36.4	0.292 ± 0.010
RS/8411/DCP	618.0 ± 10.5	0.116 ± 0.003	989.7 ± 40.5	0.244 ± 0.008	1944.8 ± 91.4	0.364 ± 0.008
8452	125.5 ± 1.5	0.055 ± 0.003	351.0 ± 4.4	0.113 ± 0.009	667.0 ± 9.7	0.311 ± 0.004
RS/8452	538.0 ± 3.7	0.125 ± 0.002	546.5 ± 6.6	0.189 ± 0.005	2046.1 ± 74.9	0.371 ± 0.009
RS/8452/DCP	862.8 ± 5.6	0.056 ± 0.002	976.6 ± 8.8	0.250 ± 0.005	2078.3 ± 25.9	0.348 ± 0.007

## Supplementary Materials

The following are available online at https://www.mdpi.com/article/10.3390/ma14216237/s1, Figure S1: The storage shear G′ and the loss shear modulus G″ for neat thermoplastic elastomers, RS/TPE blends, and for cured blends RS/TPE/DCP. Conditions of measurements: angular frequency 10 rad⋅s^−1^, temperature −20 °C. Figure S2: The storage shear G′ and the loss shear modulus G″ for neat thermoplastic elastomers, RS/TPE blends, and for cured blends RS/TPE/DCP. Conditions of measurements: angular frequency 10 rad⋅s^−1^, temperature 0 °C, Table S1: The shape fixity F and the recovery ratio RR calculated for the TPE, RS/TPE and RS/TPE/DCP blends

## References

[1] Odpady z tworzyw sztucznych i recykling w UE: Fakty i liczby. https://www.europarl.europa.eu/news/pl/headlines/society/20181212STO21610/odpady-z-tworzyw-sztucznych-i-recykling-w-ue-fakty-i-liczby.

[2] Yehia A.A. (2004). Recycling of Rubber Waste. Polym. Technol. Eng..

[3] Seghar S., Asaro L., Rolland-Monnet M., Hocine N.A. (2019). Thermo-mechanical devulcanization and recycling of rubber industry waste. Resour. Conserv. Recycl..

[4] Wang J., Xue L., Zhao B., Lin G., Jin X., Liu D., Zhu H., Yang J., Shang K. (2019). Flame Retardancy, Fire Behavior, and Flame Retardant Mechanism of Intumescent Flame Retardant EPDM Containing Ammonium Polyphosphate/Pentaerythrotol and Expandable Graphite. Materials.

[5] Movahed S.O., Ansarifar A., Zohuri G.H., Ghaneie N., Kermany Y. (2014). Devulcanization of ethylene–propylene–diene waste rubber by microwaves and chemical agents. J. Elastomers Plast..

[6] Mohaved S.O., Ansarifar A., Nezhad S.K., Atharyfar S. (2015). A novel industrial technique for recycling ethylene-propylene-diene waste rubber. Polym. Degrad. Stab..

[7] Colom X., Cañavate J., Formela K., Shadman A., Saeb M.R. (2021). Assessment of the devulcanization process of EPDM waste from roofing systems by combined thermomechanical/microwave procedures. Polym. Degrad. Stab..

[8] Maris J., Bourdon S., Brossard J.-M., Cauret L., Fontaine L., Montembault V. (2018). Mechanical recycling: Compatibilization of mixed thermoplastic wastes. Polym. Degrad. Stab..

[9] Nabil H., Ismail H., Azura A. (2013). Compounding, mechanical and morphological properties of carbon-black-filled natural rubber/recycled ethylene-propylene-diene-monomer (NR/R-EPDM) blends. Polym. Test..

[10] Nabil H., Ismail H., Azura A. (2013). Effects of virgin Ethylene–Propylene–Diene–Monomer and its preheating time on the properties of natural rubber/recycled Ethylene–Propylene–Diene–Monomer blends. Mater. Des..

[11] Nabil H., Ismail H. (2014). Enhancing the thermal stability of natural rubber/recycled ethylene–propylene–diene rubber blends by means of introducing pre-vulcanised ethylene–propylene–diene rubber and electron beam irradiation. Mater. Des..

[12] Hayeemasae N., Ismail H. (2016). Improving the Tensile Properties of Natural Rubber Compounds Containing Ground Ethylene Propylene Diene Rubber Waste by Two-stage Processing. Procedia Chem..

[13] Meng H., Li G. (2013). A review of stimuli-responsive shape memory polymer composites. Polymer.

[14] Panahi-Sarmad M., Abrisham M., Noroozi M., Amirkiai A., Dehghan P., Goodarzi V., Zahiri B. (2019). Deep focusing on the role of microstructures in shape memory properties of polymer composites: A critical review. Eur. Polym. J..

[15] Meng Q., Hu J. (2009). A review of shape memory polymer composites and blends. Compos. Part A Appl. Sci. Manuf..

[16] Verma D.K., Purohit R., Rana R., Purohit S., Patel K. (2020). Enhancement of the properties of shape memory polymers using different nano size reinforcement—A review. Mater. Today Proc..

[17] Yan C., Yang Q., Li G. (2020). A phenomenological constitutive model for semicrystalline two-way shape memory polymers. Int. J. Mech. Sci..

[18] Hoeher R., Raidt T., Rose M., Katzenberg F., Tiller J.C. (2013). Recoverable strain storage capacity of shape memory polyethylene. J. Polym. Sci. Part B: Polym. Phys..

[19] Kolesov I.S. (2008). Multiple shape-memory behavior and thermal-mechanical properties of peroxide cross-linked blends of linear and short-chain branched polyethylenes. Express Polym. Lett..

[20] Ratna D., Karger-Kocsis J. (2008). Recent advances in shape memory polymers and composites: A review. J. Mater. Sci..

[21] Meng Q., Hu J., Zhu Y., Lu J., Liu Y. (2007). Morphology, phase separation, thermal and mechanical property differences of shape memory fibres prepared by different spinning methods. Smart Mater. Struct..

[22] Razzaq M.Y., Anhalt M., Frormann L., Weidenfeller B. (2007). Mechanical spectroscopy of magnetite filled polyurethane shape memory polymers. Mater. Sci. Eng. A.

[23] Leng J., Lu H., Liu Y., Du S. Conductive nanoparticles in electro activated shape memory polymer sensor and actuator. Proceedings of the The 15th International Symposium on: Smart Structures and Materials & Nondestructive Evaluation and Health Monitoring.

[24] Lan X., Huang W.M., Liu N., Phee S., Leng J.S., Du S.Y. (2008). Improving the electrical conductivity by forming Ni powder chains in a shape-memory polymer filled with carbon black. Electroact. Polym. Actuators Devices (EAPAD).

[25] Mondal S., Hu J., Yong Z. (2006). Free volume and water vapor permeability of dense segmented polyurethane membrane. J. Membr. Sci..

[26] Hu J., Zhu Y., Huang H., Lu J. (2012). Recent advances in shape—Memory polymers: Structure, mechanism, functionality, modeling and applications. Prog. Polym. Sci..

[27] Xie T. (2011). Recent advances in polymer shape memory. Polymer.

[28] Xu C., Cui R., Chen Y., Ding J. (2019). Shape memory effect of dynamically vulcanized ethylene-propylene-diene rubber/polypropylene blends realized by in-situ compatibilization of sodium methacrylate. Compos. Part B Eng..

[29] Kolesov I., Dolynchuk O., Radusch H.J. (2015). Shape-memory behavior of cross-linked semi-crystalline polymers and their blends. eXPRESS Polym. Lett..

[30] Chatterjee T., Dey P., Nando G.B., Naskar K. (2015). Thermo-responsive shape memory polymer blends based on alpha olefin and ethylene propylene diene rubber. Polymer.

[31] Yang Q., Zheng W., Zhao W., Peng C., Ren J., Yu Q., Hu Y., Zhang X. (2019). One-way and two-way shape memory effects of a high-strain cis-1,4-polybutadiene–polyethylene copolymer based dynamic network via self-complementary quadruple hydrogen bonding. Polym. Chem..

[32] Biswas A., Aswal V.K., Maiti P. (2019). Tunable shape memory behavior of polymer with surface modification of nanoparticles. J. Colloid Interface Sci..

[33] Jagtap S., Dalvi V., Sankar K., Ratna D. (2019). Shape memory properties and unusual optical behaviour of an interpenetrating network of poly(ethylene oxide) and poly(2-hydroxyethyl methacrylate). Polym. Int..

[34] Yao Y., Luo Y., Xu Y., Wang B., Li J., Deng H., Lu H. (2018). Fabrication and characterization of auxetic shape memory composite foams. Compos. Part B Eng..

[35] Wu W., Xu C., Zheng Z., Lin B., Fu L. (2019). Strengthened, recyclable shape memory rubber films with a rigid filler nano-capillary network. J. Mater. Chem. A.

[36] Radusch H.-J., Kolesov I., Gohs U., Heinrich G. (2012). Multiple Shape-Memory Behavior of Polyethylene/Polycyclooctene Blends Cross-Linked by Electron Irradiation. Macromol. Mater. Eng..

[37] Maimaitiming A., Zhang M., Tan H., Wang M., Zhang M., Hu J.-T., Xing Z., Wu G.-Z. (2019). High-Strength Triple Shape Memory Elastomers from Radiation-Vulcanized Polyolefin Elastomer/Polypropylene Blends. ACS Appl. Polym. Mater..

[38] Cui R., Ding J., Chen Y. (2019). Magnesium acrylate induced interfacial compatibilization of EPDM/PP thermoplastic vulcanizate and shape memory behavior. Compos. Part A Appl. Sci. Manuf..

[39] Le H., Schoß M., Ilisch S., Gohs U., Heinrich G., Pham T., Radusch H.-J. (2011). CB filled EOC/EPDM blends as a shape-memory material: Manufacturing, morphology and properties. Polymer.

[40] Kumar A., Commereuc S., Verney V. (2004). Ageing of elastomers: A molecular approach based on rheological characterization. Polym. Degrad. Stab..

[41] Acharya H., Srivastava S., Bhowmick A.K. (2007). Synthesis of partially exfoliated EPDM/LDH nanocomposites by solution intercalation: Structural characterization and properties. Compos. Sci. Technol..

[42] Lipińska M., Imiela M. (2019). Morphology, rheology and curing of (ethylene-propylene elastomer/hydrogenate acrylonitrile-butadiene rubber) blends reinforced by POSS and organoclay. Polym. Test..

[43] Kader M.A., Bhowmick A.K. (2003). Rheological and viscoelastic properties of multiphase acrylic rubber/fluoroelastomer/polyacrylate blends. Polym. Eng. Sci..

