# Supplementary material for: Smart TPE Materials Based on Recycled Rubber Shred
Source: Materials (Basel). 2021 Oct 20;14(21):6237. doi: 10.3390/ma14216237 (PMC8584332; doi:10.3390/ma14216237)
Supplement: Supplementary file 1 [file materials-14-06237-s001.zip › materials-1414423-supplementary.pdf]

Smart TPE materials based on recycled rubber shred

Klaudia Toczek[1], Magdalena Lipińska[1]*, and Joanna Pietrasik[1],

[1] Lodz University of Technology, Institute of Polymer and Dye Technology, Stefanowskiego 16
90-537 Łódź, klaudia.toczek@dokt.p.lodz.pl, joanna.pietrasik@p.lodz.pl
* Correspondence: magdalena.lipinska@p.lodz.pl;

Supplementary Materials

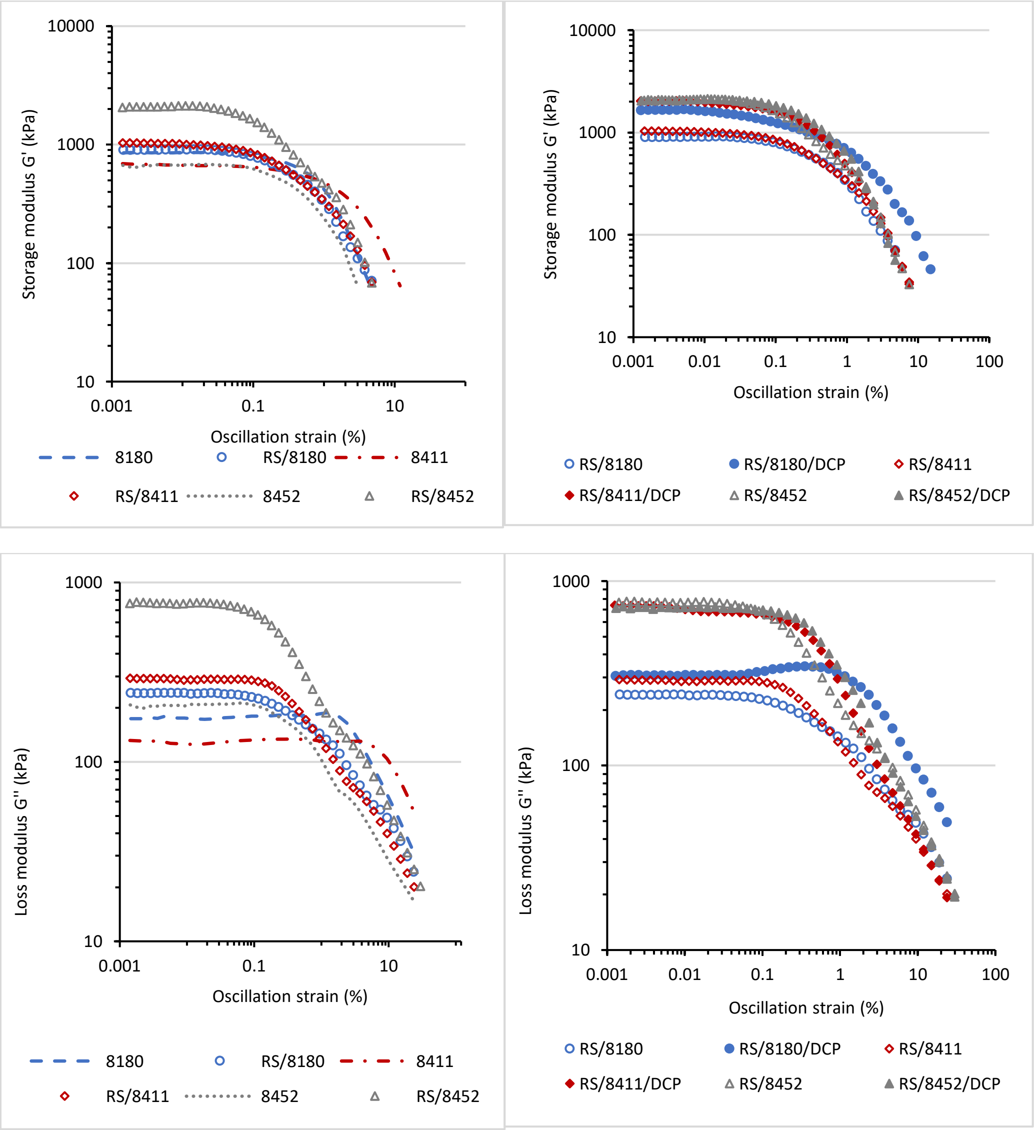

Figure S1. The storage shear G′ and the loss shear modulus G′′ for neat thermoplastic elastomers, RS/TPE blends and for cured blends RS/TPE/DCP. Conditions of measurements: angular frequency 10 rad·s^{-1}, temperature -20°C.

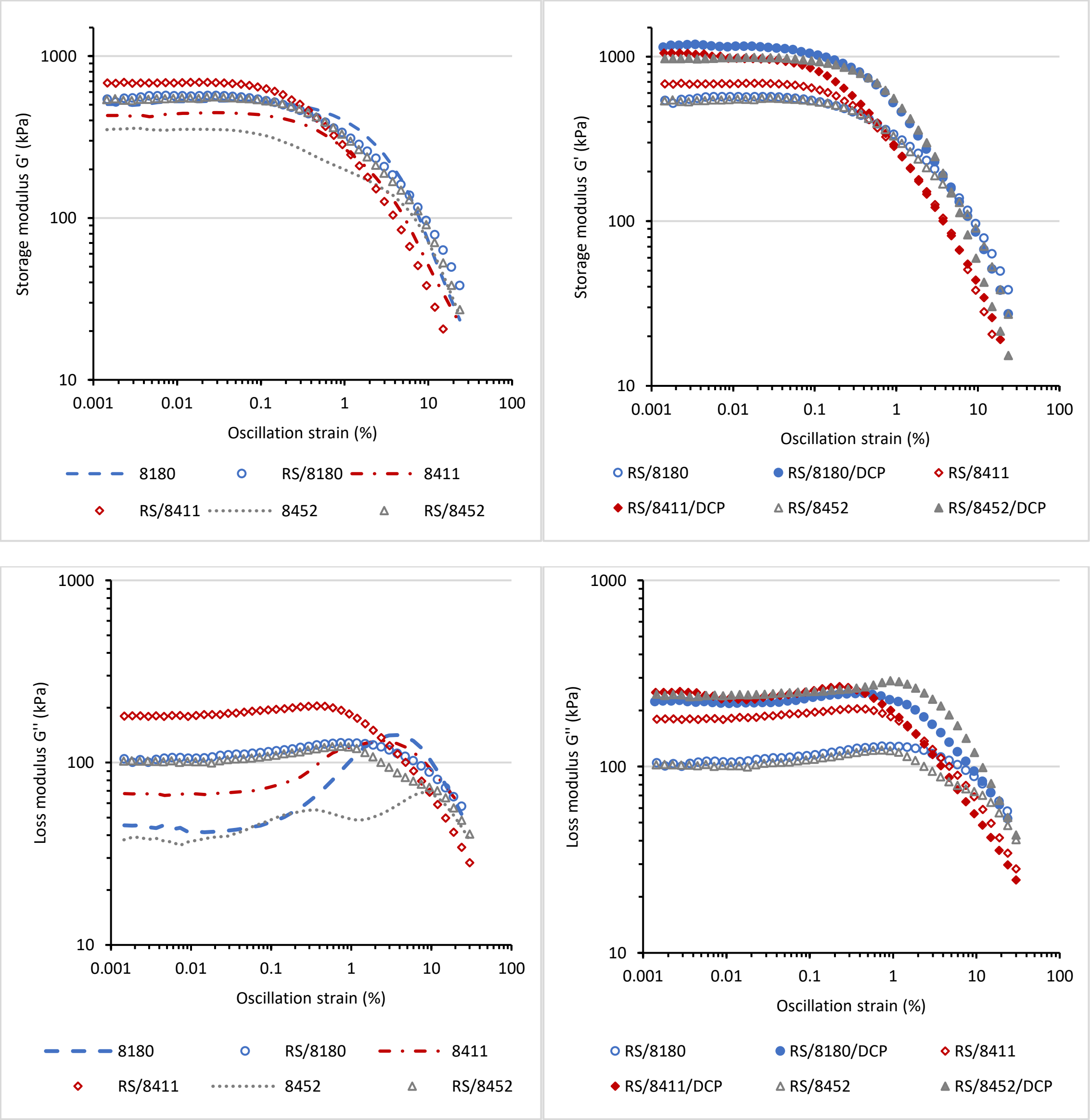

Figure S2. The storage shear G′ and the loss shear modulus G″ for neat thermoplastic elastomers, RS/TPE blends and for cured blends RS/TPE/DCP. Conditions of measurements: angular frequency 10 rad·s^{-1}, temperature 0°C.

Table S1. The shape fixity F and the recovery ratio RR calculated for the TPE, RS/TPE and RS/TPE/DCP blends.

Sample	Shape fixity F [%]	Recovery Ratio RR [%]
8411	94	91
RS/8411	86	96
RS/8411/DCP	77	100
8452	94	88
RS/8452	94	87
RS/8452/DCP	88	100
8180	98	61
RS/8180	86	64
RS/8180/DCP	84	85
